# Overcoming the Prokaryote/Eukaryote Barrier in Tuberculosis Treatment: A Prospect for the Repurposing and Use of Antiparasitic Drugs

**DOI:** 10.3390/microorganisms9112335

**Published:** 2021-11-11

**Authors:** José Manuel Ezquerra-Aznárez, Pedro E. Almeida da Silva, José A. Aínsa

**Affiliations:** 1 Departamento de Microbiología, Facultad de Medicina, Instituto de Biocomputación y Física de Sistemas Complejos (BIFI), Universidad de Zaragoza, 50009 Zaragoza, Spain; jmezquerraa@unizar.es; 2 Programa de Pós-Graduação em Ciências da Saúde, Faculdade de Medicina, Universidade Federal do Rio Grande, Rio Grande 96200-190, Brazil; 3 Instituto de Investigación Sanitaria de Aragón (IIS-Aragón), 50009 Zaragoza, Spain; 4 CIBER de Enfermedades Respiratorias (CIBERES), Instituto de Salud Carlos III, 28029 Madrid, Spain

**Keywords:** antituberculosis drugs, antiparasitic drugs, drug resistance, repurposing, drug discovery

## Abstract

Antimicrobial resistance, the so-called *silent pandemic*, is pushing industry and academia to find novel antimicrobial agents with new mechanisms of action in order to be active against susceptible and drug-resistant microorganisms. In the case of tuberculosis, the need of novel anti-tuberculosis drugs is specially challenging because of the intricate biology of its causative agent, *Mycobacterium tuberculosis*. The repurposing of medicines has arisen in recent years as a fast, low-cost, and efficient strategy to identify novel biomedical applications for already approved drugs. This review is focused on anti-parasitic drugs that have additionally demonstrated certain levels of anti-tuberculosis activity; along with this, natural products with a dual activity against parasites and against *M. tuberculosis* are discussed. A few clinical trials have tested antiparasitic drugs in tuberculosis patients, and have revealed effective dose and toxicity issues, which is consistent with the natural differences between tuberculosis and parasitic infections. However, through medicinal chemistry approaches, derivatives of drugs with anti-parasitic activity have become successful drugs for use in tuberculosis therapy. In summary, even when the repurposing of anti-parasitic drugs for tuberculosis treatment does not seem to be an easy job, it deserves attention as a potential contributor to fuel the anti-tuberculosis drug pipeline.

## 1. Introduction

Emergent and re-emergent infectious diseases caused by antimicrobial resistant microorganisms have increased drastically in recent years [1,2,3,4], being responsible for about 700,000 deaths per year worldwide. It is estimated that ~10 million deaths are caused annually, and an economic cost of $100 trillion could occur by 2050 [5,6].

Paradoxically, the development and introduction of new antimicrobials have decreased [7]. In fact, to have a strong pipeline of candidate molecules with antimicrobial activity is a huge challenge, and over the last decades, pharmaceutical companies have been progressively moving away from participating in the development of this class of drugs [8]. Indeed, the discovery and development of new antimicrobials is time consuming (it can take more than ten years) and very expensive (estimated costs range between USD 800M and USD 2Bn) [9,10,11].

In addition of requiring a huge investment, the prospect of reduced profit is another factor discouraging large companies from developing antimicrobials [10,12,13]. Several facts point in this direction: first, unlike medications used for the treatment of chronic diseases such as diabetes or hypertension, which need to be prescribed for life, most antimicrobial treatments are prescribed for a short period of time (5–14 days); second, new antimicrobials often become agents of last resort, and their use is commonly restricted; and third, the development of resistance against newly introduced antimicrobials may be fast, and hence, can compromise their use in the long term.

The lack of effective antimicrobial agents is aggravated in the case of neglected diseases such as tuberculosis (TB), caused by the bacterial pathogen *Mycobacterium tuberculosis*. TB is an age-old disease and a global public health concern at present, with about 30,000 new cases and over 4100 deaths per day worldwide [14]. Current TB treatment, consisting of a combination of several antimicrobials, is highly effective, although it is long (at least six months in the case of drug-susceptible TB) and drug-related side effects are frequent; these reasons contribute to reduced treatment adherence by patients, which ultimately may result in the selection of drug-resistant strains. Although a few new anti-TB drugs were recently introduced into the therapeutic arsenal, there is still a huge need to shorten the length of treatment, improve compatibility with anti-HIV drugs, reduce treatment-associated toxicity, and expand activity against latent and persistent bacteria. In particular, new anti-TB drugs are expected to be active for treatment of TB cases that are resistant to current anti-TB drugs, such as rifampicin-resistant TB (RR-TB), multidrug-resistant TB (MDR-TB), pre-XDR-TB (MDR-TB plus resistance to any fluoroquinolone), and XDR-TB (MDR-TB or RR-TB plus resistance to any fluoroquinolone and at least one additional Group A drug (levofloxacin, moxifloxacin, bedaquiline and linezolid)) [15,16].

In recent years, the repurposing of drugs has emerged as a reliable alternative for developing new therapies; it is estimated that around 4000 drugs that are already approved to treat several diseases are being repurposed for other therapeutic applications [17]. Repurposing an already licensed drug for a new medical application considerably saves time and capital investment in comparison with the process of developing a new drug. Drug repurposing relies on two basic concepts, one is that many drugs could have secondary biological activities that could be used for new therapeutic purposes, and the second is that different diseases could have an underlying highly similar molecular mechanism, and hence, could be targeted by the same drug [18].

In the field of infectious diseases, repurposing has been met with great expectations given the difficulties involved in developing novel antimicrobial drugs, particularly in the case of neglected diseases such as TB. Several studies have evaluated not-for-TB-approved medicines, and have identified anti-TB activity in some of them; the rationale would be to reduce time and cost to progress them as new anti-TB drugs for use in novel TB treatments [19,20].

Traditionally, the biochemical and structural differences between prokaryotic and eukaryotic cells have been widely exploited for the development of antibacterial drugs in order to increase specificity and reduce toxicity. This may have led to the conception that drugs developed for either Prokaryotes or Eukaryotes could have very limited (if any) usefulness in the other domain. However, repurposing studies are breaking down this dogma as well; for example, many medicines considered only-for-humans are finding a new role as antibacterials, and vice versa. For example, novel antimicrobial activity is being reported for ibuprofen and related drugs [21], whereas the macrolide antibiotic azithromycin is gaining use in medicine because of its wide range of biological activity [22] and the fluoroquinolone ciprofloxacin demonstrated inhibitory activity in liver cancer and other tumoral diseases [23]. In this sense, several reports of antiparasitic drugs (developed for the treatment of infections caused by protozoa or invertebrates) that demonstrated antibacterial activity captured our attention, specifically those showing anti-TB activity. Herein, we will present the state-of-the-art regarding the main antiparasitic drugs that are being considered for repurposing for TB treatment, as well as other molecules that have been reported to have both antiparasitic and anti-TB activity.

## 2. Classical Antiparasitic Drugs for Potential Anti-TB Treatment

Over the last decades, anti-TB activity has been discovered for a number of classical antiparasitic drugs. This section will describe differences in the mechanisms of action for their new potential uses in TB treatment, which are summarized in Table 1.

### 2.1. Avermectins

The avermectins are 16-membered macrocyclic lactones with broad-spectrum anthelmintic activity [24]. Their discovery was the result of a collaboration between the Kitasato Institute (led by Dr. Satoshi Õmura) and the Merck, Sharpe and Dohme Research Laboratories (led by Dr. William C. Campbell). Both researchers were awarded the Nobel Prize in Physiology or Medicine in 2015. *Streptomyces avermitilis* produces eight different avermectins (named after the word *averminous* to reflect the nature of their activity) that have subtle differences in their chemical structures but are significant in their potency [25]. Chemical modification of natural avermectins yielded ivermectin, a semi-synthetic derivative comprising no less than 80% 22,23-dihydroavermectin B_1a_ and no more than 20% of 22,23-dihydroavermectin B_1b_ [26,27]. Ivermectin is active against nematodes and several arthropods by oral, parenteral or topical routes, and is able to kill parasites either inside or outside the body. Because of its potent activity—25 times greater than all anthelmintics available at the time of their discovery—and high safety margin, it has been used in mass dosage administration campaigns to prevent onchocerciasis and lymphatic filariasis for more than three decades [28,29,30,31].

Recently, new pharmacological effects of avermectins have been identified: ivermectin has been shown to regulate glucose and cholesterol levels in diabetic mice, to suppress malignant cell proliferation in various cancer cell lines, and to inhibit viral replication in several flaviviruses. Another potential application of avermectins is as a vector control tool to prevent the transmission of protozoa that are the etiological agents of malaria, trypanosomiasis and leishmaniasis [32,33].

Among these potential applications, it was found that ivermectin and other avermectins (doramectin, moxidectin and selamectin) are active against *M. tuberculosis*, including drug-susceptible and MDR strains, a surprising finding given their lack of activity against some Gram-positive and Gram-negative bacteria [34]. However, in a posterior study, the antimicrobial activity of ivermectin against 13 members of the *M. tuberculosis* complex was not confirmed [35]. In order to sort out this discrepancy, more studies should be performed to determine the potential in vitro and/or in vivo anti-TB activity of ivermectin, either alone or in combination with other drugs used in TB treatment.

Concerning the mechanism of action, avermectins target glutamate-gated chloride channels of the ABC transporter family, increasing permeability to chloride ions and causing parasite paralysis and death [36]. Given that *M. tuberculosis* contains 14 importers and 13 exporters of the ABC transporter family [37] it was tempting to speculate on the existence of a common mechanism of action for avermectins in both bacterial and parasitic cells. However, none of the ABC transporters in *M. tuberculosis* are homologous to any of the six *Caenorhabditis elegans* glutamate-gated chloride channels [36], suggesting that the actual target of avermectins in *M. tuberculosis* is different from the target in at least this nematode, which would be consistent with the obvious differences between such organisms.

Avermectins are also active against *Mycobacterium ulcerans*, the aetiologic agent of Buruli ulcer, a necrotizing disease of the skin, subcutaneous tissue and bone. Buruli ulcer is presently the third most common mycobacterial disease in humans, after TB and leprosy [38,39,40]. More recently, it was shown that avermectins are active against a variety of non-tuberculous mycobacteria of increasing relevance in pulmonary infections concomitant with cystic fibrosis [41]. 

### 2.2. Mefloquine

The quinoline mefloquine has been used to treat and prevent chloroquine-resistant malaria for several decades. [42,43]. Subsequent studies demonstrated that mefloquine is active against *Mycobacterium avium* [44], and *M. tuberculosis* [45] in a range of concentrations between 20 and 40 µM, whereas in *Plasmodium falciparum*, mefloquine targets the 80S ribosomes [46], one of the targets for mefloquine in bacteria is the F_0_ complex of the F_0_F_1_ H^+^-ATPase, which was validated in *Streptococcus pneumoniae* [47]. This target was not experimentally validated for mycobacteria, in which early findings suggested that the target was presumably related with the cell wall structure and components [48]. In fact, a recent report identified mefloquine as an inhibitor of the MmpL3 lipid transporter in *Mycobacteroides (Mycobacterium) abscessus*, hence interfering with normal cell wall composition [49]; most probably, MmpL3 could also be the target of mefloquine in *M. tuberculosis*.

Further modifications of mefloquine have yielded scaffolds with improved potency against mycobacteria. Mefloquine-oxazolidine derivatives slightly improved the activity against pan-susceptible and MDR *M. tuberculosis* strains [50,51]. In another study on mefloquine derivatives with hybridization between the mefloquine nucleus and ethambutol, the majority of compounds showed activity against the *M. tuberculosis* pan-susceptible and MDR strains [52].

### 2.3. Niclosamide

Discovered by Bayer in 1953, niclosamide was originally developed as a drug for treating schistosomiasis. In 1982, niclosamide was approved for use in humans to treat tapeworm infection and included in the World Health Organization’s list of essential medicines [53]. 

The mode of action of niclosamide is not completely understood yet; it was traditionally believed that it inhibited oxidative phosphorylation in mitochondria and this resulted in anthelmintic activity [54]. In recent years, evidence that this drug has additional biological activities has emerged. This multifunctional drug was found to interact with different signaling pathways and biological processes. This diverse activity of niclosamide has driven several studies on its off-label use in cancer and metabolic diseases [55], also including its potential repurposing for several antiviral and antibacterial diseases [56,57,58,59].

Regarding antituberculosis activity, niclosamide inhibits the growth of the laboratory-attenuated *M. tuberculosis* strain H37Ra with a minimum inhibitory concentration (MIC) of 0.5–1 μM [60], most probably by acting as an ionophore [61]. However, at these concentrations, potential toxicity to mammalian cells was reported, thus limiting its potential as an anti-TB drug [62]. 

Coinfection between HIV and *M. tuberculosis* represents about 10% of all cases of active TB worldwide and the treatment of both infections requires therapy with multiple and compatible antibacterial and antiviral drugs. Interestingly, it was shown that niclosamide inhibits the replication of HIV, and thus, this drug could inhibit both microorganisms [63].

### 2.4. Nitazoxanide

Nitazoxanide (nitrothiazolyl) is a pro-drug that is deacetylated to tizoxanide (TIZ) in the gastrointestinal tract. Nitazoxanide is used for the treatment of infections caused by *Giardia intestinalis, Cryptosporidium parvum, Ascaris lumbricoides, Ancylostoma duodenale* and *Trichuris trichiura.* This drug also shows antibacterial activity against *Clostridium difficile* and *Helicobacter pylori*, and inhibits the replication of a broad range of viruses including respiratory syncytial virus, rotavirus, norovirus, hepatitis B, hepatitis C, dengue and yellow fever [64,65,66]. Recently, nitazoxanide was proposed for the therapy of Middle East respiratory syndrome (MERS-CoV) and Coronavirus disease 2019 (COVID-19) [67,68].

Tizoxanide was proposed to act via different mechanisms in each of the target organisms. It acts as a noncompetitive inhibitor of the pyruvate:ferredoxin oxidoreductase of protozoa and anaerobic bacteria [69]. Additional proposed targets include protozoan protein disulphide isomerases [70] and a *Giardia* nitroreductase [71]. In *C. elegans*, the target of tizoxanide is the *avr-14* subunit of a glutamate-gated chloride channel [72]. Antiviral activity of tizoxanide mostly consists of the stimulation of innate immune response (for example, by stimulating the production of interferon), and/or the interference with particular proteins in each of the viral species (for example, inhibition of hemagglutinin in influenza A virus) [73].

The antimycobacterial activity of nitazoxanide was reported against replicating and nonreplicating *M. tuberculosis* strains, affecting equally the drug-susceptible and drug-resistant strains [74,75]. This occurs through numerous mechanistic pathways, which include the disruption of *M. tuberculosis* membrane potential and pH homeostasis [64] and the inhibition of signaling pathways [76]. Interestingly, in a model mimicking caseous granuloma, nitazoxanide showed synergistic activity with rifampin, killing dormant cells in 28 to 35 days [77]. 

Recently, a randomized prospective phase II clinical trial in 30 adults with pulmonary drug-sensitive TB (ClinicalTrials.gov; Identifier: NCT02684240) showed that the administration of 1000 mg orally twice daily for 14 days lacked bactericidal activity against *M. tuberculosis*. Low plasma concentrations of nitazoxanide, below the MIC for *M. tuberculosis*, and negligible concentrations of nitazoxanide in pulmonary secretions could explain this absence of bactericidal activity [78].

### 2.5. Nitroimidazoles

Azole compounds are a large family of chemicals, some of them are being used as antifungal and antiparasitic drugs, which, in addition, have antimicrobial activity against diverse bacterial pathogens. The first active nitroimidazole described was azomycin (a 2- nitroimidazole), an antibiotic obtained from *Nocardia mesenterica* [79,80]. Different azole compound subfamilies differ in both their biological activity and mechanism of action.

Metronidazole (5-nitroimidazole) was synthetized from the scaffold of azomycin; this drug is typically used in the treatment of protozoan parasitic diseases caused by *Trichomonas vaginalis, Entamoeba histolytica*, and *Giardia lamblia*. Furthermore, metronidazole showed antibacterial activity against *Helicobacter pylori* and anaerobic bacteria [81]. Almost 30 years ago, when studying an in vitro model of dormancy, metronidazole showed antimicrobial activity against dormant *M. tuberculosis* cells [82], and the anti-TB activity of metronidazole has been confirmed in recent in vitro and ex vivo studies [83,84,85]. However, metronidazole failed to show any antimycobacterial activity in vivo, with no reduction in the bacillary burden, and, in at least one study, it even worsened lesion inflammation [86]. In addition, in other in vivo studies focusing on granuloma as a model of *M. tuberculosis* latency, metronidazole showed no anti-TB activity [87].

**Figure 1:**
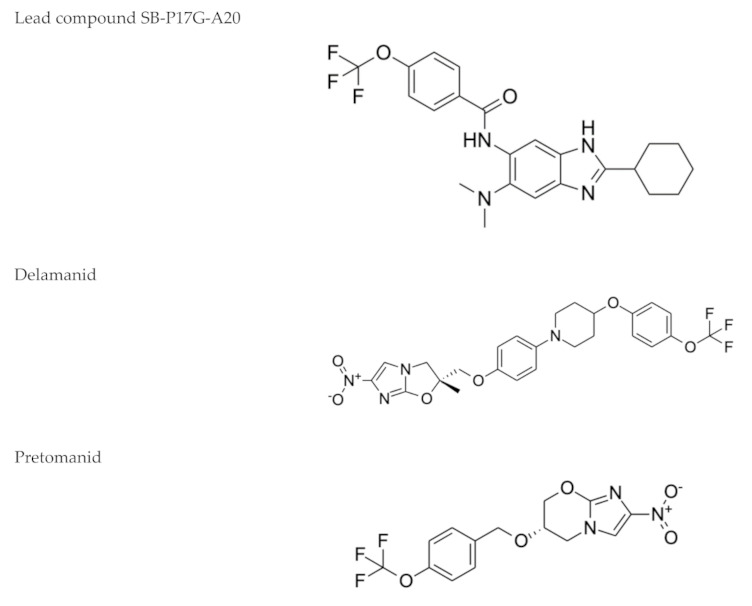
Benzimidazoles: a representative compound of this class of nitroimidazoles [89] along with delamanid and pretomanid, two drugs in clinical use against drug-resistant TB.

The benzimidazoles are another family of azole compound derivatives of benzene and imidazole; they are used in helminth infections [88], and showed antimicrobial activity against *M. tuberculosis* H37Rv as well as clinical isolates. A lead compound from this series (Figure 1) exhibited an MIC of 0.16 mg/mL and demonstrated an in vitro efficacy in the TB murine acute model of infection [89].

The target proposed for the antibacterial activity of benzimidazoles derivatives was the FtsZ protein, which was recently proposed as a target for antimicrobials [89]. Indeed, FtsZ is an important GTPase that is fundamental in bacterial cell division; in the presence of guanosine triphosphate (GTP), FtsZ polymerizes bidirectionally at the center of the bacterial cell on the inner membrane to form a highly dynamic helical structure, known as the Z-ring, which marks the future cell division site. The recruitment of several other cell-division proteins leads to contraction of the Z-ring, resulting in septum formation and cell division.

In *M. tuberculosis*, the benzimidazoles caused an enhancement of the GTPase activity of the FtsZ protein, which destabilized FtsZ assembly, leading to the efficient inhibition of FtsZ polymerization; as a consequence, cell division was arrested, but cells continued to grow, forming filaments that ultimately resulted in bacterial cell death [90].

Indeed, there are two azole compounds of the benzimidazole class that are already in clinical use in the treatment of drug-resistant TB (Figure 1): delamanid (nitro-dihydro-imidazooxazole), formerly OPC-67683, and pretomanid (nitroimidazooxazine), formerly PA-824 [14]. The proposed mechanisms of action of pretomanid and delamanid consist of the disruption of the cell wall biosynthetic machinery and inhibition of the biosynthesis of methoxy- and ketomycolates, respectively [91].

### 2.6. Pyronaridine

Pyronaridine is a potent antimalarial with chemical similarity to the aminoquinolines, a class of heterocyclic scaffolds with an amino group that is frequently found in diverse bioactive compounds, which has been widely used in China [92,93]. Pyronaridine is currently used in combination with artesunate against *P. falciparum* and *P. vivax* infections [94].

Pyronaridine showed in vitro activity (MIC = 5 μg/mL) against *M. tuberculosis*, while, in ex vivo approaches, it displayed an MIC of 12.5 μg/mL. Interestingly, it was reported that pyronaridine shows synergy with the potent anti-TB drug rifampicin, and that efflux does not play a major role in susceptibility to this drug. Further, it was proposed that interference with the metabolism of nucleic acids could be the main mechanism of *M. tuberculosis* inhibition [95].

### 2.7. Auranofin

Auranofin is a gold-containing drug, that is widely used for the treatment of rheumatoid arthritis, and was revealed to be active against *E. histolytica* at 0.5 µM after a high-throughput screening, being 10 times more active than metronidazole, the recommended treatment for amebiasis. Auranofin was effective in reducing parasite numbers in in vivo models of amebic colitis and liver abscess. Investigation of the mechanism of action of auranofin in *E. histolytica* suggested thioredoxin reductase (TrxR) as the potential target of auranofin; it was found that drug-mediated inhibition of TrxR would result in the prevention of the reduction in thioredoxin, and as a consequence, increased susceptibility of amebic trophozoites to reactive oxygen species. Interestingly, other parasites are also susceptible to auranofin killing, such as *Schistosoma mansoni*, *Trypanosoma cruzi*, *Trypanosoma brucei*, *Echinococcus granulosus*, *P. falciparum*, *G. lamblia*, *Leishmania infantum*, *Toxoplasma gondii*, and many others [96,97,98].

The antibacterial activity of auranofin was revealed when this drug was identified as being potently active against multidrug-resistant clinical isolates of *S. pneumoniae* and *Staphylococcus aureus*, both in vitro and in vivo. Auranofin was also active against other Gram-positive pathogens such as *Enterococcus faecalis*, *Enterococcus faecium*, *Enterococcus casseliflavus*, *Nocardia otitidiscaviarum*, *Streptococcus agalactiae*, and *Streptococcus pyogenes*; however, auranofin was found to have poor antimicrobial activity against Gram-negative pathogens [99]. Simultaneously, another group reported strong anti-TB activity of auranofin, with an MIC of 0.5 µg/mL against a reference strain of *M. tuberculosis* H37Rv [100], and confirmed TrxR as the target of auranofin in the tubercle bacillus.

Several clinical trials have been conducted with auranofin in recent years. First, given that auranofin PK/PD data were originated in the 1980s (when it was approved for treatment of rheumatoid arthritis), a new clinical trial studied the safety of auranofin in 15 healthy volunteers who received 6 mg/day (the standard dose for rheumatoid arthritis) during 7 days (NCT02089048; [101]). Under these conditions, auranofin was well tolerated, which supported the use of these drugs for the treatment of *E. histolytica* and *Giardia* infections, a clinical trial that is still ongoing (NCT02736968). Second, given the anti-inflammatory activity of auranofin, this drug was assayed at the same dose in a clinical trial (NCT02968927) as a host-directed adjuvant (along with standard anti-TB treatment) in TB patients; however, in this case, the treatment lasted for 112 days, and under these conditions, auranofin was not well tolerated by TB patients and showed no clinical anti-TB activity [102].

## 3. Plants as a Source of Natural Products Used in Traditional Medicine

There are some examples of natural products isolated from plants that have a wide range of biological activity, encompassing antiparasitic and anti-TB activity. Among them, we can highlight the following cases. The herb *Artemisia annua* was used for centuries as an ancient remedy for treating malaria. It contains artemisinin as a biologically active compound, which, along with its semi-synthetic derivative, artesunate, was recently described as having potential anti-TB activity [103]. Further, artemisinin showed synergistic activity with rifampicin against *M. bovis* BCG and *M. tuberculosis* H37Ra, and peroxide production increased in cells treated with both drugs [104]. *Persea americana* seeds are usually used in traditional Mexican medicine to treat several chronic, infectious diseases, and dysentery caused by helminths and amoebas. The trichloromethane extract was active against drug-susceptible and resistant strains of *M. tuberculosis.* Interestingly, ethanolic extracts were active only against *M. smegmatis*, and streptomycin and ethambutol mono-resistant strains [105]. *Cnidoscolus chayamansa*, known as Chaya, is a plant used in traditional Mexican medicine as antiprotozoal and antibacterial agent. Chloroform-methanolic (CHCl_3_:MeOH; 1:1) extracts from leaves showed antimycobacterial activity with an MIC 50 µg/mL, and hence, were used to identify the active compounds. Chemical detection of isolated compounds was performed using ^1^H- and ^13^C NMR spectra data, and moretenol and moretenyl were identified acetate as the major (but not the sole) active compounds. These had an MIC = 25 µg/mL against *M. tuberculosis* H37Rv and against four monoresistant strains of *M. tuberculosis* H37Rv, and had moderate activity against *E. histolytica* and *G. lamblia* [106].

## 4. A Special Case: The Dual Antiparasitic and Anti-TB Activity of Bacteriocin AS-48

Bacteriocins are antimicrobial peptides produced by selected bacterial species, and among them, bacteriocin AS-48, produced by *E. faecalis*, has interesting properties [107]. Bacteriocin AS-48 is a 70-amino-acid circular cationic peptide, and this unusual structure provides AS-48 with an extraordinary stability against denaturation agents such as pH and temperature, as well as a strong resistance to protease degradation [108].

Several reports described the activity of AS-48 against *Leishmania* spp [109], *T. brucei* [110], *T. cruzi* [111,112], and *M. tuberculosis* [113] and characterized its mode of action against those pathogens.

Commonly, antimicrobial peptides interact with bacterial membranes where they form pores, alter the influx and efflux of metabolites, and deplete energy; such disruptions provoke the loss of the permeability barrier. In fact, the anti-TB activity of AS-48 was clearly due to its activity on the *M. tuberculosis* membrane [113]. Interestingly, in *M. tuberculosis*, the bacteriocin AS-48 presented a clear synergism with ethambutol, one of the first-line drugs in use in the treatment of TB. Ethambutol inhibits the synthesis of arabinogalactan, thereby altering the integrity of the mycobacterial envelope, which eventually facilitates the access of AS-48 to its target, the mycobacterial membrane. The synergism between bacteriocin AS-48 and ethambutol was also observed in intracellular bacteria, which makes it interesting to consider the use of AS-48 as an adjuvant for TB treatment [113].

However, in the case of protozoan cells, although bacteriocin AS-48 may produce a certain level of depolarization in their cell membranes, its effect against other cell organelles plays a major role in the mechanism of action of AS-48.

In *Leishmania*, bacteriocin AS-48 was found to be lethal to promastigotes and, to a lesser extent, to axenic and intracellular amastigotes at low micromolar concentrations [109]. Given that only partial permeabilization of the cell membrane was observed, further investigation of additional cell targets revealed the depolarization of the single mitochondrion of *Leishmania* (hence causing a fast and dramatic energetic collapse) and the production of reactive oxygen species (ROS).

Bacteriocin AS-48 also targets the mitochondrion in *T. cruzi*, the causative agent of Chagas disease, and it is active in the low micromolecular range. In fact, it is faster in killing the trypanosomal cells and less cytotoxic than benznidazole, a drug in use against *T. cruzi* infections [112]. Given the promising results on the activity of bacteriocin AS-48 in vitro, its efficacy in vivo was tested in a mouse model of Chagas disease [111]. Bacteriocin AS-48 at 1 mg/kg was as effective as the reference drug benznidazole at 100 mg/kg in reducing the parasitaemia levels in acute *T. cruzi* infections; it also prevented immunosuppression-mediated reactivation of nested parasite cells in chronic infection, and it considerably reduced splenomegaly. This extraordinary anti-trypanosomal activity was confirmed by PCR, which indicated that 55% of organs in AS-48-treated mice were parasite free.

The protozoan *T. brucei*, the causative agent of sleeping sickness in humans and nagana in cattle, is extremely susceptible to nanomolar concentrations of AS-48, even lower than those required to kill the most susceptible bacteria *L. monocytogenes*; moreover, AS-48 was more active against *T. brucei* than other antimicrobial peptides that killed this protozoan at micromolar concentrations. The high activity of AS-48, together with the low toxicity to eukaryotic cell lines, resulted in a large selectivity index (>10^4^) for this bacteriocin. The mode of action of bacteriocin AS-48 in *T. brucei* is notably different from those described above. In this species, AS-48 targets cellular compartments rather than affecting the plasma membrane or the mitochondrion [110]. Upon its interaction with a highly abundant protein on the surface of *T. brucei*, bacteriocin AS-48 is internalized through clathrin-mediated endocytosis. Once inside the cells, it provokes major changes in the ultrastructure of the parasite, due to the appearance of myelin-like structures and double-membrane autophagic vacuoles that ultimately result in the induction of autophagy and cell death.

## 5. Discussion

Despite major structural differences between eukaryotic and prokaryotic cells, which have been widely exploited for the sake of the selectivity of antibacterial agents, in recent years, we have witnessed a number of examples of molecules having significant activity against bacterial cells and also against specific types of eukaryotic cells, notably those of protozoa and helminths. Our interest has focused on those products with activity against *M. tuberculosis*, which was responsible for the highest number of deaths per year in the world due to a single pathogen until the COVID-19 pandemic, starting in 2020, broke this deadly record [14].

Even though it is extremely urgent to find new drugs for treating TB, the success has been very limited, with only three novel drugs (bedaquiline, delamanid and pretomanid) being approved in the last fifty years. The scarcity of chemical scaffolds that are active against mycobacteria is one of the reasons behind this poor outcome. However, the findings reported in this review open a promising alternative. The development of new anti-TB drugs through the re-utilization (repurposing or repositioning) of drugs active against parasites can increase the portfolio of therapeutic alternatives for TB. Several antiparasitic drugs, classic or in pre-clinical steps of development, have shown activity in vitro and ex vivo against *M. tuberculosis*, although very few clinical trials have explored this possibility yet.

Antiparasitic drugs with activity against *M. tuberculosis*, even when their anti-TB activity may not be sufficient or achievable in therapy of humans, may still be the starting point for the development of new derivatives with greater efficacy and less toxicity. In fact, we can find a clear example of this scenario in the case of metronidazole: as we mentioned above, in an in vitro dormancy model, metronidazole and other azoles such as benzimidazole derivatives showed activity against *M. tuberculosis*, although, in in vivo infection models, metronidazole completely lacked anti-TB activity. Hopefully, this knowledge opened the door to the development of novel azole derivatives (delamanid and pretomanid) that were recently approved for the treatment of TB.

Discovery of a novel scaffold through such promising repurposing approaches is just a first step in the broadening of our armamentarium against *M. tuberculosis* and other neglected pathogens. However, this strategy comes with various limitations that must be assessed. First, and most importantly, the mode of action of the candidate molecules must be determined against *M. tuberculosis*. Given the differences between *M. tuberculosis* and the variety of organisms encompassed within the term *parasite*, it can be expected that the new activity is based upon the promiscuity of the molecule to different targets rather than on both species sharing a common target [18]. We reviewed three examples of this scenario: the avermectins target an ABC type of glutamate-gated chloride channel in the nematode cells, whereas, in *M. tuberculosis*, we speculate on the possibility of a different mechanism of action given that no clear homologue of such an ABC transporter could be identified; second, mefloquine, which targets the 80S ribosomes in *P. falciparum* and targets the MmpL3 lipid transporter in mycobacteria; and third, the bacteriocin AS-48, which targets the membrane in bacterial cells, whereas, in protozoa such as *Leishmania* and *T. cruzi*, it depolarizes the membrane of the mitochondrion. In the latter case, we could argue that targeting the mitochondrion could be considered as having *a similar* mechanism of action as in bacterial cells, given the proven prokaryotic origin of this organelle.

Adjusting therapeutic doses for such naturally different pathogens and infections is another limitation behind the repurposing strategies of anti-infective compounds. In this way, the presence of the bacterial cell wall hinders the interaction between antimicrobials and their targets. *M. tuberculosis* possesses a highly complex cell wall, which comprises the inner membrane, a core of covalently linked peptidoglycan, arabinogalactan and mycolic acids, a leaflet of extractable lipids, and a capsule [114]. Such a structure, combined with the expression of multidrug efflux pumps, renders *M. tuberculosis* virtually impermeable to any molecule. Hence, relevant anti-TB activity is generally observed at concentrations of compounds higher than those needed for killing protozoans. In some cases, those therapeutic concentrations needed for killing TB with an antiparasitic drug are impossible to achieve in vivo. The case of nitazoxanide illustrates this situation. Previous studies reported MICs of nitazoxanide of 12.3 µg/mL for *M. tuberculosis*, and this was expected to be reached after twice daily administrations of 1000 mg of nitazoxanide. However, due to its high binding to plasma proteins, the average nitazoxanide concentration in plasma was around 10.2 µg/mL, which is clearly below the concentration needed for inhibiting the growth of *M. tuberculosis* [78].

As long as higher doses are needed to achieve therapeutic effects, the risk of drug-associated toxicity and side effects increases. This is particularly relevant in the case of *M. tuberculosis* infections, because the WHO-recommended treatment for drug-susceptible cases of TB lasts for six months, which excludes potential active drugs having tolerable side effects upon short-term treatment, but this was unfortunately found not to be appropriate for such long treatments. Unfortunately, this was the case of auranofin, a drug with a promising anti-TB activity, targeting a new pathway in *M. tuberculosis*, and with a record of safety for its use in rheumatoid arthritis, which was, however, not well tolerated after a four months of treatment in TB patients. In the case of niclosamide, this drug is generally well tolerated for the treatment of tapeworm, possibly because it does not need to be absorbed to be effective since this parasite lives in the human gut; however, since toxicity was reported in mammalian cell lines, it is probable that a long-lasting systemic or pulmonary delivery of niclosamide for treating TB could result in serious side effects [62]. The potential toxicity of avermectins has been well documented and although these drugs are generally considered as highly safe for humans, with only transient and mild to moderate side effects, more severe toxic effects could be expected from their use at higher concentrations or for long periods of time [115]. When toxicity becomes a relevant issue, two alternatives can be explored. First, such hypothetical new active but toxic scaffolds identified through repurposing approaches could enter medicinal chemistry programs to improve their selectivity towards *M. tuberculosis* and new validated druggable targets, and this would result in reduced doses and, consequently, reduced potential toxicity. A second alternative comes from synergistic combinations; as was discussed in the case of bacteriocin AS-48, its synergy with ethambutol allows a 32-fold reduction in the dose required to kill *M. tuberculosis* [113] and, consequently, to minimize any potential toxic effect on mammalian cells.

Finally, we can foresee some positive aspects for a future use of common drugs for the treatment of TB and other infectious diseases. Co-existing infections among bacteria, viruses and parasites are common, and the epidemiological impact, as well as the clinical evolution of a given infectious disease, is related to other concomitant infections. Furthermore, treating two or more diseases at the same time in a given patient involves several pharmacokinetic, pharmacodynamic and pharmacogenetic challenges [116]. Parasitic infections are highly prevalent in the developing world, with a high rate of geographic overlap with regions that have a high prevalence of TB [117]. Although the relationship between TB and parasitic infections is still poorly understood, intestinal infections with helminths and protozoa were shown to have a negative association with clinical outcomes for TB [117]. Treatment with anti-TB drugs that also act as antiparasitics could improve the clinical outcomes of TB while reducing the burden of parasitic infections.

## 6. Conclusions and Future Perspectives

Several reports have demonstrated that some antiparasitic drugs have secondary biological activities, and this property can be exploited for potential repurposing for other medical applications. In particular, for the drugs discussed in this review, their additional biological activity has melted away the barrier between drugs for eukaryotes and drugs for prokaryotes, and has positioned them in the challenging field of potential anti-TB drugs. Although the provision of new molecular scaffolds for TB therapy through repurposing approaches is a shortcut in drug development, there are still many issues to be solved, such as adjusting the therapeutic dose, avoiding toxicity, and elucidating the mechanism of action, among others. Natural differences between parasites and *M. tuberculosis*, and between infections caused by either pathogen, are clearly behind this. Hopefully, this study reported cases in which, for example, medicinal chemistry was decisive in terms of sorting out such difficulties by producing derivatives of original antiparasitic drugs with improved properties for TB therapy. In the future, it is expected that other disciplines such as nanotechnology and pharmacogenomics will also contribute in this direction.

## Figures and Tables

**Table 1:** Main antiparasitic drugs that are being studied to be repurposed for TB treatment.

Drug Name, Class and Structure ^1^	Current Use	Rationale for TB Repurposing	Possible TB Target/ Mode of Action
**Avermectins** (Ivermectin B1a) 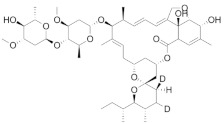	Prevention of onchocerciasis and lymphatic filariasis	Active against *M. tuberculosis*, including M/XDR isolates (MIC = 3–6 µg/mL)	Not yet determined
**Mefloquine** 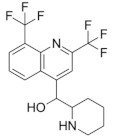	Chloroquine-resistant malaria	Active against *M. tuberculosis* (MIC = 20–40 µM)	MmpL3
**Niclosamide** 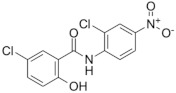	Tapeworm infections	Active against *M. tuberculosis* H37R A(MIC = 0.5–1 µM)	Ionophore
**Nitazoxanide** 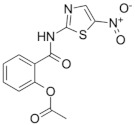	Infections caused by *Giardia intestinalis*, *Cryptosporidium parvum*, *Ascaris lumbricoides*, *Ancylostoma duodenale* and *Trichuris trichiura*	Active against replicating and nonreplicating *M. tuberculosis*	Disruption of membrane potential and pH homeostasis. Inhibition of signaling pathways
**Nitroimidazoles** (Metronidazole) 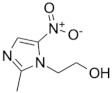	Helminth infections (benzimidazoles)	Active against *M. tuberculosis* in in vitro and ex vivo assays. Benzimidazoles show activity in murine models	FtsZ (benzimidazoles); mycolic acid biosynthesis (delamanid and pretomanid)
**Pyronaridine** 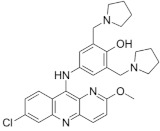	Malaria	Active against *M. tuberculosis* in in vitro (MIC = 5 µg/mL) and ex vivo (MIC = 12.5 µg/mL) assays	Interference with nucleic acid metabolism
**Auranofin** 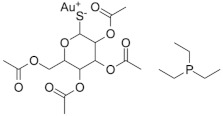	Amebiasis	Active against *M. tuberculosis* (MIC = 0.5 µg/mL)	Thioredoxin reductase TrxR

^1^ The structures of the compounds were obtained by ChemDraw using Computed Descriptors: Canonical or Isomeric SMILES deposited in PubChem.
